# A Machine Learning Model for Food Source Attribution of *Listeria monocytogenes*

**DOI:** 10.3390/pathogens11060691

**Published:** 2022-06-16

**Authors:** Collins K. Tanui, Edmund O. Benefo, Shraddha Karanth, Abani K. Pradhan

**Affiliations:** 1 Department of Nutrition and Food Science, University of Maryland, College Park, MD 20742, USA; ctanui@umd.edu (C.K.T.); ebenefo@umd.edu (E.O.B.); skm@umd.edu (S.K.); 2 Center for Food Safety and Security Systems, University of Maryland, College Park, MD 20742, USA

**Keywords:** *Listeria monocytogenes*, food source attribution, whole-genome sequencing, machine learning, predictive modeling

## Abstract

Despite its low morbidity, listeriosis has a high mortality rate due to the severity of its clinical manifestations. The source of human listeriosis is often unclear. In this study, we investigate the ability of machine learning to predict the food source from which clinical *Listeria monocytogenes* isolates originated. Four machine learning classification algorithms were trained on core genome multilocus sequence typing data of 1212 *L. monocytogenes* isolates from various food sources. The average accuracies of random forest, support vector machine radial kernel, stochastic gradient boosting, and logit boost were found to be 0.72, 0.61, 0.7, and 0.73, respectively. Logit boost showed the best performance and was used in model testing on 154 *L. monocytogenes* clinical isolates. The model attributed 17.5 % of human clinical cases to dairy, 32.5% to fruits, 14.3% to leafy greens, 9.7% to meat, 4.6% to poultry, and 18.8% to vegetables. The final model also provided us with genetic features that were predictive of specific sources. Thus, this combination of genomic data and machine learning-based models can greatly enhance our ability to track *L. monocytogenes* from different food sources.

## 1. Introduction

Foodborne illnesses affect approximately 48 million people in the United States every year, resulting in an estimated 128,000 hospitalizations and 3000 deaths [1]. About a fifth (approximately 9.4 million) of these can be attributed to known pathogens [2,3]. In most outbreak investigations, disease etiology is linked to individual foods, which enables public health authorities, regulatory agencies, and the food industry to identify potential points of contamination. Foodborne outbreak data can also be used to identify emerging food safety concerns and evaluate the effectiveness of foodborne illness prevention programs [4]. Foods of animal origin, fruits, and vegetables are usually implicated in most foodborne outbreaks [2,5,6]. Common pathogenic bacteria responsible for foodborne outbreaks include *Listeria monocytogenes*, *Campylobacter*, *Salmonella*, and Shiga toxin-producing *Escherichia coli*, among others [3,7]. 

*L. monocytogenes* causes serious illness only in a small percentage of healthy people. According to the United States Centers for Disease Control and Prevention (CDC), about 1600 people get listeriosis annually, and about 260 succumb to it [8]. Even though the number of listeriosis cases is lower than that of other foodborne illnesses, the disease burden of this pathogen is higher because of the serious nature of the disease when vulnerable groups are affected [8,9]. Listeriosis is ranked third among the causes of foodborne illness-associated deaths in the United States, causing nearly 19% of these deaths [10]. People who are at risk for listeriosis include pregnant women, the elderly, people with weakened immune systems, and newborns [10]. 

Food animals, particularly ruminants, can get infected with *L. monocytogenes*, making them potential zoonotic reservoirs of this pathogen [11,12]. Human infections are rarely related to exposure to infected animals or fomites from agricultural environments. However, animal-derived food products eaten raw or undercooked and refrigerated ready-to-eat (RTE) foods stored for long periods are known to cause listeriosis in humans [13,14]. 

Fresh produce is another food group that is gradually becoming a major route of human exposure to *L. monocytogenes* [15,16]. Unlike other foodborne pathogens, *L. monocytogenes* can thrive under alternative (i.e., non-ideal) conditions, such as low moisture, high salt concentration, and refrigeration temperature environments [17]. Since 2010, over 85 multistate outbreaks with confirmed etiology have been attributed to fresh produce in the United States [8]. Cross-contamination within the supply chain, improper storage temperatures during distribution, and improper food preparation practices are some of the frequently implicated contributors to these events. 

Food source attribution is the process of estimating the most common food categories responsible for illnesses caused by specific pathogens [18,19]. Source attribution enables the identification of the relative contributions of different food sources to the occurrence of foodborne illnesses [20,21]. To achieve this, several sources of data are required including epidemiological, laboratory-, and outbreak-related data [22,23]. Unraveling the sources of foodborne illness is vital to identifying strategies to improve food safety along the entire food production and supply chain [19,24].

Multilocus sequence typing (MLST) [25,26] has been the preferred method for population genetic analyses, with the results usually corroborating epidemiological findings [26,27]. This molecular technique has been used to monitor changes in food microbial reservoirs, particularly those changes that arise as a result of interventions targeting the food chain and public health [26,28,29,30]. According to a prior study [26], core genome MLST (cgMLST) and allelic variations can be used to differentiate isolates and link them to food sources in source attribution studies. To decrease the prevalence of foodborne diseases and minimize microbial contamination in food, effective monitoring of the distribution and occurrence of foodborne pathogens is essential. It is worth noting that foodborne pathogens are resilient; this means that they can adapt genetically and phenotypically to the extreme conditions found in host and non-host systems, which allows them to survive and proliferate under these conditions [3,31,32]. These changes could be particularly informative towards identifying the basis of pathogen adaptation to, and survival and virulence in, host systems, as well as their response to safe food handling practices in the industry and by consumers. Therefore, a careful analysis of these changes could, in the long run, help develop methods and practices to reduce the risk of foodborne outbreaks. 

In recent years, there has been a growing interest in analyzing genome sequencing data using artificial intelligence (AI), particularly machine learning (ML) [33]. Mechanistic model-based methods are aimed at formulating simplified mathematical models to explain various phenomena by carefully examining, analyzing, and identifying patterns in relevant data [34]. On the other hand, ML focuses on ‘learning’ from relevant patterns in data, and using this information to make predictions [35,36]. Basically, by exploring and identifying patterns in data, ML can be used in the classification, regression, or clustering of data to draw meaningful inferences from the same. Genome sequencing information, coupled with machine learning, has been used to predict the risk of listeriosis in humans [37], the host specificity of *S. enterica* and *E. coli* [38], and host disease severity based on *S. enterica* gene presence/absence [36,39], and in the source attribution of *S.* Typhimurium [26]. With the increase in usage of genome sequencing for exploratory and integrated surveillance activities, generating massive amounts of data, as well as standardization of data collection activities (providing us with useful metadata and other useful information), machine learning and big data analytical tools become the need of the hour to provide a better understanding and improvement of current knowledge in foodborne disease epidemiology.

This study aimed at developing a ML-based model for source attribution of human listeriosis by analyzing *L. monocytogenes* core genomes. The model was based on cgMLST profiles from clinical *L. monocytogenes* isolates and isolates from dairy, fruits, leafy greens, meat, poultry, seafood, and vegetables.

## 2. Results 

### 2.1. Predictive Model 

We developed a supervised machine learning model to predict the possible source of human listeriosis cases based on allelic variations in *L. monocytogenes* isolates from foods. Of the 1748 *L. monocytogenes* core genes, 1012 genes were removed due to zero or near-zero variance (see Section 4.3.1), leaving 736 genes that were used in the model.

The performance of random forest, logit boost, stochastic gradient boosting, and support vector machine radial kernel models were compared using the average accuracies obtained from 10 iterations applying 10-fold cross-validation. All four models performed well with accuracies between 0.614 and 0.732, and Kappa values between 0.530 and 0.657 (Table 1).

The performance of logit boost (0.732), random forest (0.722), and stochastic gradient boosting (0.701) did not differ significantly from one another. However, these three models performed significantly better than support vector machine. Receiver operating characteristic (ROC) curves were generated for the different models. The areas under the curve (AUC) for logit boost, random forest, gradient boosting machine, and support vector machine were 0.865, 0.805, 0.822, and 0.820, respectively. Logit boost had the highest accuracy and AUC among the models considered and was selected for further analysis.This selection was also substantiated by the Kappa value for the logit boost model (0.654), suggesting a ‘substantial’ agreement between the observed and predicted classes [40] or a ‘fair to good’ agreement based on Fleiss’s criteria [41]. 

Confusion matrix statistics of all train-test models are presented in Supplementary Table S1. Logit boost, the best performing model, had a specificity > 0.90 for all food sources, and sensitivity > 0.7 for most food sources, except leafy greens (0.548), meat (0.484), and poultry (0.447). The low sensitivity observed in leafy greens, meat, and poultry could be due to the smaller sample size in these categories as compared to the other categories. In the future, with the availability of an increased number of samples, especially in the less dominant classes, it may be possible to increase the sensitivity of the model for these classes. Other methods to potentially further improve the sensitivity of classifiers may include the use of resampling techniques and cost-sensitive learning approaches in future studies. 

### 2.2. Source Attribution of Human Listeriosis Cases 

**Figure 1:**
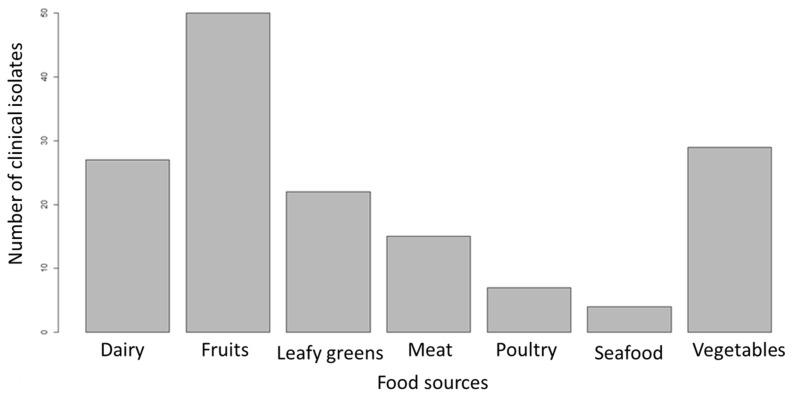
Predicted sources of clinical *L. monocytogenes* isolates.

We trained a new model using logit boost on the complete feature-reduced data set (Supplementary Table S2). This model predicted the probable food sources of each of the 154 clinical *L. monocytogenes* isolates. The model predicted that 32.5% of the clinical isolates originated from fruits, 18.8% from vegetables, and 17.5%, 14.3%, 9.7%, 4.6%, and 2.6% from dairy, leafy greens, meat, poultry, and seafood, respectively (Figure 1).

### 2.3. Important Predictor Genes

Twenty of the most important genes were analyzed in isolates from different sources of food using logit boost, and their functional classes were determined based on an extensive literature survey. These genes allow us to identify microbial genetic patterns associated with each food source. According to Table 2, genes associated with survival, adaptation, and stress response were mainly found to be important in isolates from fresh produce, meat, and poultry. Additionally, two-component transcriptional regulators and virulence genes were found in isolates from fresh produce. However, some significant predictors/genes remained undefined in isolates from all food sources. 

## 3. Discussion

### 3.1. Source Attribution Model

A major prerequisite for improving public health is preventing the emergence and spread of foodborne diseases. Source attribution models help link sporadic human cases of a specific foodborne illness to its food source. With the increasing usage of genome sequencing technologies, it is possible to identify genetic patterns indicative of the food source of pathogens. Recently, machine learning models have been used to identify molecular markers from foodborne pathogens linked with different hosts/phenotypes, which could be used to trace the source of human infections [26,36,37,39]. In the current study, we investigated the potential of machine learning to predict the food source origins of bacterial strains isolated from human cases of listeriosis using machine learning analyses of cgMLST data. Our machine learning model was able to recognize patterns in the complex dataset and use this information to predict the source of human listeriosis isolates. These patterns were based on variations in the genetic composition of *L*. *monocytogenes* isolated from different food sources. Furthermore, we identified allele variations that can be considered as being important predictors for this traceback process.

Due to the rapid adoption of genome sequencing technologies such as whole-genome sequencing (WGS) in food microbiology and public safety, new source attribution modeling approaches incorporating molecular information have been emerging. These methods generate comprehensive genomic data, providing critical insight into the transmission patterns of several major foodborne diseases, including listeriosis [42,43]. Here, we developed a machine learning-based source attribution model using the core genomes of 1212 *L. monocytogenes* isolates from different food sources. In our study, we have employed a high cutoff for the cgMLST allele calls. As a result, missing values in the cgMLST profiles can range from very low to very high, as seen in a prior study conducted by Kshirsagar and colleagues (2012). Another potential reason for missing data could be that some of the isolates may not possess the loci altogether. However, for successful modeling using machine learning techniques, complete data is essential, since missing values impact the overall effectiveness of the model(s). This issue can be overcome by imputing missing values [44]. In this study, missing allelic values in the food and clinical isolates were imputed. The total number of allele calls imputed in the isolates ranged from <1–78%, based on data completeness, which is consistent with that seen in a previous study [45]. As a result, our model performance was considerably improved, as seen in the model statistics. As shown in Table 1, logit boost was the best performing model (accuracy = 0.732, 95% confidence interval (CI) 0.665–0.760; Kappa = 0.654). A recent study [46] used a similar method to trace the source of salmonellosis, using random forest to determine the possible source of zoonotic outbreaks [46].

After testing a number of ML approaches, logit boost was used in source attribution in this study. Our model predicted that most of the listeriosis cases may have originated from produce (fruits 32.5%; vegetables 18.8%; leafy greens 14.3%), 9.7% from meat, 4.6% from poultry, and 2.6% from seafood. Several studies have reported listeriosis outbreaks linked to the consumption of meats, dairy products, fresh produce, and seafood contaminated with *L*. *monocytogenes* [6,8,15,16,46,47,48,49,50,51,52,53]. Contamination of food sources may occur at any point in the production chain due to many factors [16,54]. The primary source of contamination or cross-contamination has been identified as originating from the farm environment, machinery, and staff [55,56,57]. This, however, is contingent on food handlers’ level of hygienic practice. To avoid cross-contamination or recontamination during production and along the supply chain, food handlers must maintain personal hygiene and properly sanitize touch surfaces and production lines [58]. Finally, optimal cooking temperatures for specific food products should be considered during preparations [57], and temperatures in storage refrigerators should be properly monitored to prevent pathogens from growing, especially as *L. monocytogenes* stress response mechanisms allow it to survive non-thermal hurdle interventions [59,60,61].

### 3.2. Important Top Twenty Predictor Genes

Identifying the origin, also known as attribution, of microbial isolates is important within the realm of infectious diseases, specifically those caused due to direct or indirect contact with food or food sources. Prior efforts in this direction have focused on comparing the genotype, and its associated markers, of the isolate of interest with those seen in source populations [62,63,64,65,66,67]. Thus, it stands to reason that the increase in usage of genome sequencing methods in various aspects of food and outbreak surveillance should provide researchers with a wealth of features to analyze for source attribution purposes. However, the addition of such a large number of features can overwhelm current models due to the sheer scale of data and the amount of computation time added [62,63,64,65,66,67]. 

Prior studies have shown how such issues can be addressed by analyzing these complex data sets with ensemble machine learning classification [26,68]. In addition to accurate predictions, machine learning models can identify features that have the best prediction potential. Using our logit boost model, we identified 20 of the 736 *L. monocytogenes* genes that were the most important predictors for the attribution of listeriosis to different food sources. Our results (Table 2) showed that most of these genes were associated with *L. monocytogenes’* survival and stress response.

*L. monocytogenes* can adapt to, and survive, a wide range of stress conditions, including extremes of pH, temperature, and salt concentrations, which makes it problematic for food producers who rely on pathogen response to these stresses for food preservation. Stress tolerance in *L. monocytogenes* can be partially explained by the presence of the general stress response genes; transcription of these genes during host contamination provides homeostatic and protective functions to cope with the stress [11,69]. The *recR* gene, which encodes recombination protein and is involved in DNA repair, transcriptional genes *degU*, *cesR*, and *mlrA*, which encode putative response regulators that control many virulence factors, transporters *lmo2215* and *srlA*, and many genes coding for hypothetical proteins (*lmo2401*, *lmo2577*, *lmo2348*, *lmo0623*, *lmo0635*, *lmo2658*, and *lmo1425*) were identified as being important in association with the food sources studied. The putative DegU response regulator is a pleiotropic regulator involved in microbial motility at low temperatures [70]. This indicates the relevance of DegU in the current model, as most of the food sources studied are refrigerated or frozen to extend their shelf life—DegU may enable the survival of *L. monocytogenes* at low temperatures, contributing to its persistence in these foods, subsequently leading to listeriosis in humans who consume the contaminated food.

Furthermore, the presence of the response regulator CesR and the histidine protein kinase CesK, which is encoded by the gene downstream from *cesR*, indicates *L. monocytogenes’* ability to tolerate ethanol and antibiotics of the beta-lactam family (which act on the microbial cell wall) [71]. These genes may also enhance the persistence of *L. monocytogenes* in different food sources. Eight out of the twenty most important genes were hypothetical genes, which is in line with the findings of prior studies [36,39]. Thus, future studies involving the characterization of each gene to understand its importance in *L. monocytogenes* adaptation and stress response along the food supply chain are warranted.

In the current study, we explored the use of machine learning in source attribution based on *L*. *monocytogenes* WGS data. Without a doubt, pathogens with food safety implications are not fully understood biologically, such as the relationship between specific infections and their sources. Our study shows that incorporating machine learning, surveillance, and monitoring infrastructures such as the National Antimicrobial Resistance Monitoring System and GenomeTrakr (which have been generating and uploading copious amounts of foodborne pathogen genomes) will allow researchers to draw a meaningful conclusion from genome-informed datasets. Machine learning is presumably positioned to address many of the current challenges in the food safety industry. By using machine learning, it may be possible to uncover patterns in WGS data that are not easily gleaned from traditional methods. Thus, it may be possible to solve difficult problems in food source attribution using genomic data.

In conclusion, supervised machine learning was effective in attributing food sources to listeriosis clinical cases based on WGS data. Inferring genetic information from pathogen genotypes often proves crucial for biological inference. Source attribution of *L*. *monocytogenes* infections allows food industry professionals, data managers, epidemiologists, microbiologists, and bioinformaticians to tailor their practices to prevent the spread of foodborne pathogens. It also enables healthcare professionals to more efficiently use resources to contain the survival and proliferation of pathogens at the source. As genomic data becomes more widely available, WGS serves as a cost-effective method for public health surveillance. With the availability of hundreds of thousands of genomes of foodborne pathogens and evolutionary relationships rapidly being determined, sequencing information can be used for prediction purposes when combined with useful isolate metadata, particularly in the food safety domain. One limitation of this study was that, while an ideal validation scenario would involve validating the model on a new data set (such as an unused subsample of data during model training), all of our data were used for model development due to the limited number of samples. However, in the future, the model can be validated on new data as it becomes available. 

## 4. Materials and Methods

### 4.1. Data Description 

*L. Monocytogenes* isolates included in this study (n = 1366) were sampled from the National Centers for Biotechnology Information’s (NCBI) Pathogen Detection database and included 154 isolates from human listeriosis patients and 1212 isolates from food sources, including dairy (197), fruits (302), leafy greens (115), meat (119), poultry (119), seafood (145), and vegetables (215) (Supplementary Table S3). The included *L. monocytogenes* isolates were extracted from food and clinical sources as part of integrated surveillance and were previously sequenced (using different platforms such as Illumina HiSeq, NextSeq, or MiSeq). A simple random sampling of 10 to 60% of all available isolates from each source was performed, based on the availability of relevant metadata (such as location, isolation source, source type, and Interagency Food Safety Analytics Collaboration (IFSAC) category), which served as isolate inclusion criteria. The clinical isolates selected were also sampled from publicly available sequences, and as such were not epidemiologically associated with specific outbreaks.

### 4.2. Bioinformatics Analysis

Input for the source attribution model was generated from all sequences within the data set by running cgMLST. The Enterobase scheme was used to obtain cgMLST [72,73] in BioNumerics v.7.6 (Applied Maths, Sint Martens Latem, Belgium). *L. monocytogenes* has 1748 core genes, with each loci having several allele variations [72,73]. The cgMLST allele calls were accepted when the strains had a core genome coverage of more than 95% (1661) of the 1748 core genome alleles, and detection of mixed sequence alleles of less than 50 alleles. In some cases, BioNumerics may fail to call an allele as a result of stop codons, indels, and other factors in the genome sequence, resulting in missing values in the cgMLST profile. In such cases, we used the missForest package in R (version 4.1.2) to impute the missing values. In the missForest package, missing values are imputed using random forest trained on the observed data to predict the missing values [55]. 

### 4.3. Source Attribution Modeling

Machine learning algorithms were used to predict the food source of a given strain isolated from human listeriosis cases based on allelic variations found in the core genes of *L. monocytogenes* isolated from food sources (dairy, fruits, green leafy vegetables, meat, poultry, and seafood). In this study, we used supervised machine learning classification models. Here, our models learned patterns in the allelic variations of the *L. monocytogenes* isolates from food sources. Modeling was carried out in R (v. 4.1.2, R Core Team, 2021; Vienna, Austria) using the caret package [74,75].

#### 4.3.1. Feature Reduction

The core genome of *L. monocytogenes* consists of 1748 loci [72,73]. Feature reduction was performed using the nearZeroVar (near zero variance) function in the caret package in R to remove some features (genes). NearZeroVar identifies features that have a single unique value or have very few unique values relative to the number of samples, or when the frequency ratio (frequency of most frequent value divided by the frequency of second most frequent value) is large [74]. This is because retaining these redundant features that provide no useful details to distinguish between the food sources may only increase computation time and model complexity.

#### 4.3.2. Machine Learning

Our feature-reduced cgMLST data was randomly split into a training set (70%) and a testing set (30%). Four machine learning algorithms–random forest, logit boost, stochastic gradient boosting, and support vector machine radial kernel–have been successfully applied in studies analyzing WGS data [36,37,39,76] and were therefore used in training our data. We used 10-fold cross-validation, which randomly partitions the training data set into 10 equal folds—nine folds used for model training and one fold to estimate model performance—to obtain the model with the best performance. This procedure was repeated until 10 models had been trained, each using unique training and testing folds. The default hyperparameter grid (in the R package *caret*) was employed to search for optimal tuning parameters for all four algorithms. The final tuning parameters utilized for the models, based on the best-fit Kappa values, were: LB (31 nIter), RF (38 mtry), GBM (150 n.trees, 3 interaction depth, 0.1 shrinkage, and 10 n.minobsinnode), and SVMR (0.002580397 sigma and 1 C).

The developed models were evaluated against the testing set and the performance of the models was assessed based on the Kappa value, model accuracy, and other confusion matrix statistics. The accuracy was calculated from the models’ ability to correctly classify the testing data set. The Kappa value is a statistic that compares the model accuracy (observed accuracy) with the expected accuracy [77]. It shows the agreement between predicted and actual classes and is especially important in highly unbalanced data where the accuracy can be misleading. We performed 10 iterations of model training and testing and selected the algorithm that achieved the highest average accuracy as the best algorithm for further analysis.

A final model was developed by training the best-performing algorithm on the complete feature-reduced cgMLST data. This was run to allow the algorithm to learn as much as possible from the variability in the complete data set. This approach has been successfully implemented by [26] and has been identified as the best approach for a predictive model. The best performing model was then used to predict the probable food sources of each of the 154 clinical *L. monocytogenes* isolates.

## Figures and Tables

**Table 1:** Models performance from 10 iterations of random forest, support vector machine radial kernel, stochastic gradient boosting, and logit boost models.

Models	Accuracy	95% CI	Kappa
Logit boost	0.732 ^a^	0.665–0.760	0.654
Random forest	0.722 ^a^	0.667–0.776	0.657
Stochastic gradient boosting	0.701 ^a^	0.645–0.745	0.633
Support vector machine	0.614 ^b^	0.569–0.671	0.530

Values under the Accuracy column with different superscripts are significantly different (*p* < 0.05).

**Table 2:** Twenty putative genes sorted by maximum importance across the food sources.

Loci	Gene	Protein Name	Dairy	Fruits	Leafy Greens	Meat	Poultry	Seafood	Vegetables
lmo2702	*recR*	Recombination protein RecR	0.6653	0.5945	0.6925	0.8315	0.7212	0.6219	0.6653
lmo2401	*lmo2401*	Hypothetical protein	0.7017	0.663	0.6997	0.8231	0.7664	0.663	0.7017
lmo2615	*rpsE*	30S ribosomal protein S5	0.6873	0.5786	0.708	0.8199	0.7465	0.6081	0.6873
lmo2577	*lmo2577*	Hypothetical protein	0.7066	0.6611	0.6809	0.808	0.7851	0.6611	0.7066
lmo1501	*lmo1501*	Hypothetical protein	0.6925	0.6014	0.6839	0.8022	0.716	0.6374	0.6925
lmo1933	*folE*	GTP cyclohydrolase 1	0.577	0.6111	0.599	0.8012	0.7435	0.627	0.6111
lmo2215	*lmo2215*	Similar to ABC transporter (ATP-binding protein)	0.692	0.6473	0.6633	0.7988	0.72	0.6473	0.692
lmo0821	*lmo0821*	Hypothetical protein	0.6641	0.6641	0.7076	0.7979	0.7461	0.6641	0.657
lmo1715	*lmo1715*	Methyltransferase	0.674	0.6314	0.6612	0.7963	0.7371	0.6314	0.674
lmo2515	*degU*	NarL family, response regulator DegU	0.6923	0.6482	0.6759	0.7952	0.7781	0.6482	0.6923
lmo0625	*lmo0625*	Putative lipase/acylhydrolase	0.6548	0.6242	0.6813	0.7945	0.743	0.6242	0.6548
lmo0544	*srlA*	PTS sorbitol transporter subunit IIC	0.7125	0.6483	0.7073	0.7928	0.7713	0.6483	0.7125
lmo2728	*mlrA*	Transcriptional regulator, MerR family protein	0.62	0.6322	0.6294	0.7909	0.6994	0.6041	0.6322
lmo2348	*lmo2348*	Amino acid ABC transporter permease	0.6776	0.6673	0.681	0.7901	0.7512	0.6673	0.6776
lmo2422	*cesR*	Two-component response regulator	0.6988	0.6498	0.6574	0.7883	0.7307	0.6498	0.6988
lmo0623	*lmo0623*	Hypothetical protein	0.6382	0.6382	0.6382	0.7877	0.7026	0.6382	0.6307
lmo0635	*lmo0635*	Hypothetical protein	0.6715	0.6715	0.7008	0.7872	0.744	0.6715	0.656
lmo2658	*lmo2658*	Hypothetical protein	0.5621	0.5409	0.5969	0.7859	0.6298	0.5644	0.5621
lmo0611	*azoR1*	Azoreductase	0.626	0.6511	0.7853	0.7737	0.626	0.626	0.6511
lmo1425	*lmo1425*	Hypothetical protein	0.7079	0.651	0.6755	0.7852	0.7607	0.651	0.7079

Note: The numbers represent importance based on the accuracies of source prediction by each feature (genes). These values are the area under the receiver operating characteristic curve (AUC-ROC) determined from source-specific sensitivities and specificities (Supplementary Tables S1 and S2).

## Data Availability

Not applicable.

## Supplementary Materials

The following supporting information can be downloaded at: https://www.mdpi.com/article/10.3390/pathogens11060691/s1, Table S1: Statistics of the confusion matrices for LB, RF, GBM, and SVMR (train and test models); Table S2: Statistics of the confusion matrices for the final (best performing) model (LB); Table S3: *Listeria monocytogenes* isolates (indicated by their BioSample numbers) from food sources and clinical samples used to generate cgMLST profiles.
