# Supplementary material for: A Machine Learning Model for Food Source Attribution of Listeria monocytogenes
Source: Pathogens. 2022 Jun 16;11(6):691. doi: 10.3390/pathogens11060691 (PMC9230378; doi:10.3390/pathogens11060691)
Supplement: Supplementary file 1 [file pathogens-11-00691-s001.zip › Supplementary Table S1.pdf]

Supplementary Table S1. Statistics of the confusion matrices for LB, RF, GBM, and SVMR (train and test models).

LB							
	**Dairy**	**Fruits**	**Leafy greens**	**Meat**	**Poultry**	**Seafood**	**Vegetables**
**Sensitivity**	0.780	0.898	0.548	0.484	0.447	0.750	0.702
**Specificity**	0.938	0.914	0.961	0.952	0.957	0.969	0.970
**Pos. Pred. Value**	0.708	0.793	0.567	0.484	0.548	0.750	0.839
**Neg. Pred. Value**	0.957	0.961	0.958	0.952	0.937	0.969	0.935
**Accuracy**	0.859	0.906	0.755	0.718	0.702	0.860	0.836

RF							
	**Dairy**	**Fruits**	**Leafy greens**	**Meat**	**Poultry**	**Seafood**	**Vegetables**
**Sensitivity**	0.830	0.835	0.565	0.500	0.458	0.696	0.733
**Specificity**	0.937	0.879	0.964	0.972	0.952	0.980	0.974
**Pos. Pred. Value**	0.759	0.755	0.591	0.611	0.478	0.762	0.846
**Neg. Pred. Value**	0.959	0.923	0.960	0.957	0.948	0.972	0.949
**Accuracy**	0.884	0.857	0.765	0.736	0.705	0.838	0.854

GBM							
	**Dairy**	**Fruits**	**Leafy greens**	**Meat**	**Poultry**	**Seafood**	**Vegetables**
**Sensitivity**	0.797	0.878	0.452	0.581	0.421	0.625	0.716
**Specificity**	0.954	0.899	0.976	0.952	0.936	0.960	0.963
**Pos. Pred. Value**	0.771	0.761	0.636	0.529	0.432	0.658	0.814
**Neg. Pred. Value**	0.960	0.952	0.950	0.961	0.933	0.954	0.938
**Accuracy**	0.875	0.888	0.714	0.766	0.678	0.792	0.840

SVMR							
	**Dairy**	**Fruits**	**Leafy greens**	**Meat**	**Poultry**	**Seafood**	**Vegetables**
**Sensitivity**	0.729	0.878	0.161	0.323	0.263	0.600	0.716
**Specificity**	0.866	0.823	1.000	0.994	0.979	0.944	0.923
**Pos. Pred. Value**	0.512	0.647	1.000	0.833	0.588	0.571	0.676
**Neg. Pred. Value**	0.943	0.948	0.928	0.940	0.919	0.950	0.935
**Accuracy**	0.797	0.850	0.581	0.658	0.621	0.772	0.820

Pos. pred.: positive predictive; Neg. pred.: negative predictive.
