# Supplementary material for: A Machine Learning Model for Food Source Attribution of Listeria monocytogenes
Source: Pathogens. 2022 Jun 16;11(6):691. doi: 10.3390/pathogens11060691 (PMC9230378; doi:10.3390/pathogens11060691)
Supplement: Supplementary file 1 [file pathogens-11-00691-s001.zip › Supplementary Table S2.pdf]

Supplementary Table S2. Statistics of the confusion matrices for the final (best performing) model (LB).

LB							
	**Dairy**	**Fruits**	**Leafy greens**	**Meat**	**Poultry**	**Seafood**	**Vegetables**
**Sensitivity**	0.918	0.966	0.800	0.667	0.636	0.933	0.960
**Specificity**	0.970	0.953	1.000	0.996	0.996	0.976	0.974
**Pos. Pred. Value**	0.865	0.904	1.000	0.933	0.933	0.824	0.889
**Neg. Pred. Value**	0.983	0.984	0.985	0.974	0.970	0.992	0.991
**Accuracy**	0.944	0.960	0.900	0.831	0.816	0.955	0.967

Pos. pred.: positive predictive; Neg. pred.: negative predictive.
