# Supplementary material for: A Machine Learning Model for Food Source Attribution of Listeria monocytogenes
Source: Pathogens. 2022 Jun 16;11(6):691. doi: 10.3390/pathogens11060691 (PMC9230378; doi:10.3390/pathogens11060691)
Supplement: Supplementary file 1 [file pathogens-11-00691-s001.zip › Supplementary Table S3.pdf]

Supplementary Table S3. ***Listeria monocytogenes*** **isolates (indicated by their BioSample numbers) from food sources and clinical samples used to generate cgMLST profiles.**

Dairy		Fruits				Leafy greens		Meat	
SAMN05179419	SAMN04547037	SAMN16826415	SAMN03278639	SAMN02937460	SAMN10083734	SAMN21582197	SAMN01939119	SAMN13898671	SAMN16816525
SAMN02923525	SAMN04575417	SAMN15717930	SAMN02999666	SAMN02937456	SAMN15970345	SAMN21582205	SAMN01939119	SAMN13898660	SAMN16816786
SAMN02923524	SAMN04575421	SAMN16826096	SAMN02999665	SAMN02937455	SAMN10026580	SAMN21582200	SAMN01816214	SAMN13898627	SAMN17188240
SAMN02923526	SAMN04575440	SAMN16826889	SAMN03287037	SAMN02937448	SAMN10083718	SAMN21582199	SAMN01816207	SAMN13898669	SAMN17188248
SAMN02923527	SAMN04506949	SAMN16825957	SAMN03287038	SAMN02937447	SAMN15913265	SAMN19716581	SAMN01939127	SAMN13898677	SAMN18800556
SAMN02923663	SAMN04506944	SAMN16826920	SAMN05058062	SAMN02937446		SAMN19716580	SAMN03787619	SAMN13898664	SAMN18800705
SAMN02922153	SAMN04506947	SAMN06330455	SAMN05058067	SAMN02937445		SAMN01816208	SAMN04244459	SAMN13898688	SAMN16814138
SAMN02922152	SAMN04539453	SAMN03702255	SAMN05058070	SAMN02937465		SAMN03329999	SAMN04244460	SAMN13898640	SAMN18800706
SAMN02922151	SAMN04575433	SAMN04929605	SAMN05058073	SAMN02937466		SAMN03329998	SAMN04244461	SAMN13898639	SAMN18800642
SAMN02922150	SAMN04506954	SAMN04301827	SAMN05058580	SAMN02937469		SAMN02582706	SAMN04244462	SAMN13898697	SAMN16816955
SAMN02347441	SAMN04506952	SAMN04929616	SAMN05058583	SAMN02937470		SAMN01939120	SAMN04244463	SAMN13898673	SAMN17188178
SAMN03339953	SAMN04506953	SAMN02567781	SAMN05058586	SAMN02937464		SAMN04480762	SAMN02569969	SAMN06446248	SAMN16822078
SAMN02922906	SAMN04506942	SAMN04929612	SAMN05058729	SAMN02937467		SAMN04480763	SAMN02567858	SAMN05191816	SAMN16825972
SAMN02922901	SAMN04539455	SAMN04347223	SAMN05058733	SAMN02937468		SAMN05213462	SAMN02567856	SAMN07409542	SAMN17188231
SAMN02921367	SAMN04506963	SAMN04347219	SAMN05058736	SAMN02922106		SAMN02709403	SAMN02567857	SAMN04992025	SAMN17188774
SAMN02922940	SAMN04506957	SAMN04301828	SAMN15717907	SAMN04929620		SAMN05721602	SAMN02567866	SAMN21220912	SAMN17188778
SAMN02998027	SAMN04506937	SAMN02923601	SAMN15717939	SAMN02953954		SAMN05721610		SAMN21220914	SAMN16825943
SAMN13569405	SAMN04575430	SAMN02934656	SAMN02902857	SAMN02953957		SAMN05721609		SAMN21220905	SAMN16813131
SAMN13747397	SAMN04539445	SAMN02934655	SAMN02902859	SAMN02953956		SAMN05721543		SAMN21220915	SAMN03357032
SAMN13747395	SAMN04539444	SAMN02850636	SAMN03024043	SAMN02953953		SAMN05721611		SAMN21220911	SAMN03357082
SAMN06958595	SAMN04506943	SAMN02850635	SAMN03024041	SAMN02924020		SAMN04450321		SAMN21220913	
SAMN06958594	SAMN04539447	SAMN07554887	SAMN03024044	SAMN02924025		SAMN04450323		SAMN22160592	
SAMN02713589	SAMN08287555	SAMN06555603	SAMN03024033	SAMN02924028		SAMN04450325		SAMN22160580	
SAMN02318984	SAMN08287520	SAMN06555609	SAMN03024039	SAMN03135513		SAMN04450327		SAMN05179365	
SAMN02318986	SAMN04506959	SAMN06555608	SAMN03024031	SAMN03135515		SAMN05721545		SAMN03267996	
SAMN02318985	SAMN04506948	SAMN06555611	SAMN02902858	SAMN03135514		SAMN05577724		SAMN05179363	
SAMN06620527	SAMN04547029	SAMN06555604	SAMN02902860	SAMN03135539		SAMN21580652		SAMN06113187	
SAMN10397001	SAMN04539448	SAMN06555605	SAMN08274613	SAMN06645599		SAMN23523467		SAMN06113186	
SAMN06620523	SAMN04539449	SAMN06555607	SAMN08274625	SAMN03135516		SAMN23523469		SAMN06113184	
SAMN02666872	SAMN04575407	SAMN06555610	SAMN08274646	SAMN03135517		SAMN23523468		SAMN06113185	
SAMN02666874	SAMN04575423	SAMN06555606	SAMN08274628	SAMN03135537		SAMN10352998		SAMN15638749	
SAMN02666876	SAMN04575408	SAMN06555601	SAMN08274650	SAMN06645600		SAMN08097737		SAMN15638748	
SAMN02666880	SAMN04575439	SAMN06555602	SAMN03897566	SAMN03135511		SAMN08097731		SAMN11318567	
SAMN02666875	SAMN04506950	SAMN02887168	SAMN02709385	SAMN03135512		SAMN12262208		SAMN11318570	
SAMN13286390	SAMN04547030	SAMN03260235	SAMN02709397	SAMN06645597		SAMN12230436		SAMN11371679	
SAMN13286395	SAMN04506962	SAMN02887167	SAMN02709396	SAMN03135510		SAMN02569968		SAMN11371677	
SAMN13286389	SAMN04506955	SAMN16826909	SAMN02709362	SAMN06645598		SAMN11491410		SAMN11318753	
SAMN13286391	SAMN04539452	SAMN03097579	SAMN03097642	SAMN04943311		SAMN11491435		SAMN03736712	
SAMN13286394	SAMN04575383	SAMN03097576	SAMN03285264	SAMN02991391		SAMN02567864		SAMN12822632	
SAMN13286392	SAMN04575415	SAMN03097580	SAMN03285263	SAMN02991381		SAMN12230499		SAMN12783937	
SAMN03736764	SAMN04575425	SAMN03097578	SAMN02567794	SAMN02991387		SAMN12230515		SAMN11310117	
SAMN05521541	SAMN04506960	SAMN06645596	SAMN03135520	SAMN02990454		SAMN02912058		SAMN11310118	
SAMN03135524	SAMN04506961	SAMN06645595	SAMN03135518	SAMN02991383		SAMN02912060		SAMN11310160	
SAMN03135523	SAMN04506958	SAMN02924006	SAMN03135535	SAMN02990456		SAMN01939116		SAMN04600027	
SAMN03736730	SAMN04522131	SAMN02924007	SAMN03135536	SAMN02991378		SAMN07560063		SAMN04600026	
SAMN03736767	SAMN04522130	SAMN16826816	SAMN03135521	SAMN02990452		SAMN07560054		SAMN04600041	
SAMN07177538	SAMN24693939	SAMN03702791	SAMN03135522	SAMN02990456		SAMN07560057		SAMN04600054	
SAMN04146103	SAMN24693938	SAMN03702786	SAMN03135519	SAMN02991390		SAMN05179355		SAMN03168667	
SAMN10756631	SAMN13961219	SAMN03702785	SAMN03135529	SAMN02991379		SAMN08163920		SAMN03168669	
SAMN19354052	SAMN18093947	SAMN03702789	SAMN03135530	SAMN02991386		SAMN08163922		SAMN16813072	
SAMN19354215	SAMN19368296	SAMN03702790	SAMN03135534	SAMN02990453		SAMN06669436		SAMN16814580	
SAMN19353849	SAMN19488895	SAMN03702799	SAMN03135533	SAMN02991388		SAMN08163914		SAMN16815324	
SAMN19353708	SAMN19696758	SAMN03702800	SAMN03135532	SAMN02991382		SAMN04117176		SAMN16816031	
SAMN02937514	SAMN07552046	SAMN03702802	SAMN03015665	SAMN02991390		SAMN04192096		SAMN16816789	
SAMN02937515	SAMN07552031	SAMN03702805	SAMN02709380	SAMN03023705		SAMN04192105		SAMN16817018	
SAMN06240079	SAMN07552032	SAMN03702792	SAMN02709383	SAMN03023706		SAMN02567871		SAMN16817770	
SAMN03218398	SAMN07552045	SAMN03702801	SAMN02709378	SAMN03023707		SAMN04102353		SAMN16822410	
SAMN03218401	SAMN15072558	SAMN03702795	SAMN02709360	SAMN03023708		SAMN04102354		SAMN16822405	
SAMN03218400	SAMN12547590	SAMN03702796	SAMN02850679	SAMN03002777		SAMN03983448		SAMN16825949	
SAMN03218403	SAMN12547587	SAMN03702803	SAMN02850678	SAMN03002782		SAMN02567860		SAMN16825974	
SAMN03218399	SAMN12642147	SAMN03702804	SAMN02709382	SAMN03002788		SAMN02567859		SAMN16826074	
SAMN02383870	SAMN12642333	SAMN03702797	SAMN02709407	SAMN03002785		SAMN01939125		SAMN16826319	
SAMN02769767	SAMN14096434	SAMN04146147	SAMN02709381	SAMN03002785		SAMN02850685		SAMN16826313	
SAMN02769766	SAMN14096444	SAMN03085716	SAMN02709379	SAMN03002788		SAMN02567863		SAMN17188781	
SAMN02922665	SAMN06005706	SAMN03085717	SAMN02953935	SAMN03002789		SAMN03656762		SAMN16813067	
SAMN02922666	SAMN03165750	SAMN02570799	SAMN02953937	SAMN03002784		SAMN02922246		SAMN16813085	
SAMN15969076	SAMN05191814	SAMN02570800	SAMN02953934	SAMN03002786		SAMN02922247		SAMN16813125	
SAMN04358689	SAMN06005707	SAMN07258003	SAMN03285164	SAMN03002787		SAMN02419859		SAMN16814156	
SAMN04358690	SAMN07571685	SAMN06645608	SAMN03285163	SAMN02991374		SAMN02922242		SAMN16814174	
SAMN03285311	SAMN07571688	SAMN03135538	SAMN03285135	SAMN02990446		SAMN02922243		SAMN16814310	
SAMN04358691	SAMN12286920	SAMN06240119	SAMN03285134	SAMN02990445		SAMN02922244		SAMN16814349	
SAMN05462169	SAMN12286909	SAMN06240121	SAMN03285136	SAMN02990449		SAMN02922245		SAMN16814381	
SAMN04386791	SAMN07444219	SAMN06240118	SAMN03285132	SAMN02990450		SAMN10393075		SAMN16814579	
SAMN04386792	SAMN07571689	SAMN06240120	SAMN03285133	SAMN02991375		SAMN10393070		SAMN16814447	
SAMN11478342	SAMN07571687	SAMN23523484	SAMN03285131	SAMN02990447		SAMN10393033		SAMN16815203	
SAMN03703776	SAMN07571683	SAMN23523483	SAMN03285204	SAMN02991377		SAMN10393030		SAMN16815201	
SAMN03703780	SAMN12684842	SAMN06240078	SAMN03285203	SAMN02991392		SAMN10393045		SAMN16815285	
SAMN03703788	SAMN12684872	SAMN06240055	SAMN03285228	SAMN02953500		SAMN10393067		SAMN16815322	
SAMN03703779	SAMN06005712	SAMN22401822	SAMN03285227	SAMN02953501		SAMN10393018		SAMN16816105	
SAMN03770170	SAMN06760093	SAMN26214649	SAMN12035822	SAMN03462163		SAMN10393044		SAMN16817027	
SAMN03770154	SAMN02769709	SAMN26218028	SAMN04929609	SAMN03462161		SAMN10393021		SAMN16817753	
SAMN06242534	SAMN03277781	SAMN03287226	SAMN03285194	SAMN03218268		SAMN10393032		SAMN16818242	
SAMN06242522	SAMN03736704	SAMN03287236	SAMN03285193	SAMN06645656		SAMN10389891		SAMN16826055	
SAMN06242535	SAMN03285200	SAMN03287229	SAMN07519901	SAMN06645603		SAMN10389887		SAMN16826317	
SAMN06242514	SAMN03431388	SAMN03287230	SAMN07520046	SAMN02924077		SAMN10389889		SAMN16826435	
SAMN06242536	SAMN03431389	SAMN03287232	SAMN03135503	SAMN02924071		SAMN10389896		SAMN16826905	
SAMN05821180	SAMN03431390	SAMN03287225	SAMN03135505	SAMN07554890		SAMN03760076		SAMN17188242	
SAMN03465571	SAMN03431392	SAMN03287224	SAMN03135507	SAMN02924074		SAMN01939115		SAMN18800683	
SAMN03465570	SAMN03431391	SAMN03287231	SAMN03135504	SAMN07554894		SAMN06890560		SAMN16826459	
SAMN03465569	SAMN02769710	SAMN03287234	SAMN03135506	SAMN02924075		SAMN06477572		SAMN16816187	
SAMN03465573	SAMN02769679	SAMN03287237	SAMN07520045	SAMN02924076		SAMN06477565		SAMN15508345	
SAMN03465572	SAMN02921771	SAMN02797538	SAMN07520051	SAMN07554891		SAMN06475417		SAMN16814357	
SAMN03465574	SAMN02921772	SAMN02797539	SAMN07520052	SAMN07554893		SAMN06476216		SAMN16814443	
SAMN09984273	SAMN02566980	SAMN01939109	SAMN03135508	SAMN16826025		SAMN06476209		SAMN16817769	
SAMN09986740	SAMN02566981	SAMN03278634	SAMN03135509	SAMN16825969		SAMN06476236		SAMN16814440	
SAMN26027220	SAMN02769680	SAMN03278637	SAMN02937458	SAMN16826247		SAMN10460979		SAMN16826268	
SAMN04884978	SAMN02769681	SAMN03278638	SAMN02937461	SAMN15913297		SAMN10461334		SAMN16946575	
SAMN04884979	SAMN02769682	SAMN03278635	SAMN02937457	SAMN15913258		SAMN03495904		SAMN16816091	
SAMN04506966		SAMN03278636	SAMN02937459	SAMN10083736		SAMN01939119		SAMN16816335	

Poultry		Seafood		Vegetables			Clinical Samples		
SAMN02922173	SAMN16815204	SAMN02937501	SAMN10977210	SAMN21582201	SAMN15913301	SAMN15970348	SAMN09756851	SAMN03354313	
SAMN02922174	SAMN16812961	SAMN02937503	SAMN04146069	SAMN06555523	SAMN09953956	SAMN15913260	SAMN09756858	SAMN11268677	
SAMN02922175	SAMN16813075	SAMN02937504	SAMN03736731	SAMN06555522	SAMN10026609	SAMN15913281	SAMN09756873	SAMN06240103	
SAMN06477613	SAMN16815286	SAMN02923311	SAMN03736755	SAMN03787623	SAMN10026594	SAMN15913262	SAMN07831756	SAMN07831580	
SAMN13047656	SAMN16816124	SAMN02923310	SAMN03736726	SAMN03787625	SAMN10026591	SAMN15970354	SAMN09756840	SAMN06240099	
SAMN13047990	SAMN16816128	SAMN02923308	SAMN03736729	SAMN04902934	SAMN10026589	SAMN10083716	SAMN06240095	SAMN01816124	
SAMN04858632	SAMN16817021	SAMN02923309	SAMN04146134	SAMN06457941	SAMN10026607	SAMN10083723	SAMN05371170	SAMN09756952	
SAMN13429586	SAMN16821849	SAMN02923298	SAMN04146133	SAMN03702235	SAMN10083714	SAMN10083738	SAMN05371174	SAMN09756936	
SAMN17987503	SAMN16825945	SAMN02923504	SAMN04146131	SAMN03702236	SAMN10083730	SAMN15913273	SAMN05371160	SAMN02769702	
SAMN08097585	SAMN16825970	SAMN02923499	SAMN04146121	SAMN03702237	SAMN15913259	SAMN15913267	SAMN05371166	SAMN03277722	
SAMN03277808	SAMN16826450	SAMN02923500	SAMN04146114	SAMN03702238	SAMN15913294	SAMN10083733	SAMN05371167	SAMN03277677	
SAMN03277808	SAMN16826439	SAMN04893488	SAMN04146115	SAMN04916636	SAMN15913254	SAMN15913264	SAMN05371164	SAMN06240109	
SAMN05179362	SAMN16826886	SAMN04893493	SAMN04146116	SAMN04916637	SAMN15970335	SAMN15913263	SAMN07831627	SAMN08134177	
SAMN03277699	SAMN16814733	SAMN04893494	SAMN05900811	SAMN04916638	SAMN10026592	SAMN15913300	SAMN09756853	SAMN14400383	
SAMN03277810	SAMN16822140	SAMN04893495	SAMN04146135	SAMN04916641	SAMN10026582	SAMN09953964	SAMN07831629	SAMN14400408	
SAMN03277666	SAMN16826253	SAMN04914047	SAMN04146032	SAMN05967956	SAMN10026581	SAMN10083717	SAMN07831760	SAMN03277635	
SAMN03277667	SAMN16814448	SAMN04914048	SAMN03736756	SAMN04640315	SAMN10083724	SAMN10026605	SAMN11268698	SAMN03354302	
SAMN03277702	SAMN16814600	SAMN04914049	SAMN03736757	SAMN04640301	SAMN10083727		SAMN09756903	SAMN03277636	
SAMN04487283	SAMN16815196	SAMN04914050	SAMN05521497	SAMN04640307	SAMN15913290		SAMN11268717	SAMN03277671	
SAMN04146078	SAMN16817024	SAMN04914060	SAMN05900814	SAMN04640310	SAMN15913279		SAMN02381991	SAMN14400405	
SAMN04146093		SAMN02921927	SAMN05521570	SAMN08040165	SAMN10083713		SAMN02399607	SAMN12610330	
SAMN04146075		SAMN02921928	SAMN11803304	SAMN06256026	SAMN10083731		SAMN02389107	SAMN12610365	
SAMN07791951		SAMN02921930	SAMN05233165	SAMN04148255	SAMN15913274		SAMN02381990	SAMN02769705	
SAMN05384842		SAMN02921929	SAMN04544913	SAMN05371084	SAMN20248553		SAMN02402290	SAMN02769705	
SAMN07312510		SAMN02922005	SAMN05233164	SAMN02921341	SAMN09953943		SAMN07831758	SAMN08134167	
SAMN10977201		SAMN02922085	SAMN05233166	SAMN02921339	SAMN09953959		SAMN07831759	SAMN06240102	
SAMN05521551		SAMN02922004	SAMN05233169	SAMN02921338	SAMN09953958		SAMN09756862	SAMN03277767	
SAMN05521545		SAMN02922087	SAMN05233170	SAMN02921340	SAMN10026608		SAMN09756860	SAMN08134294	
SAMN04487243		SAMN02922006	SAMN03736710	SAMN16791495	SAMN10026610		SAMN09756859	SAMN08134288	
SAMN04487240		SAMN02922007	SAMN03736709	SAMN16792401	SAMN15913255		SAMN09756842	SAMN03277646	
SAMN05900843		SAMN02400355	SAMN03736766	SAMN12056316	SAMN15970356		SAMN09756861	SAMN03277647	
SAMN07823453		SAMN02400356	SAMN03274505	SAMN12056313	SAMN20248558		SAMN14400383	SAMN03277643	
SAMN03736702		SAMN02921358	SAMN03274504	SAMN04155494	SAMN15913299		SAMN14400408	SAMN03277644	
SAMN04487279		SAMN02922612	SAMN02937472	SAMN04991858	SAMN10083721		SAMN03277635	SAMN03354292	
SAMN04487255		SAMN02921433	SAMN02921899	SAMN04155489	SAMN09953933		SAMN03354302	SAMN02769773	
SAMN04487242		SAMN02922084	SAMN02921902	SAMN04155490	SAMN09953954		SAMN03277636	SAMN08134202	
SAMN02921631		SAMN02921981	SAMN02921789	SAMN11491398	SAMN09953961		SAMN03277671	SAMN03354291	
SAMN02921632		SAMN02923414	SAMN06200231	SAMN02677677	SAMN15913252		SAMN14400405	SAMN03277645	
SAMN03277793		SAMN03198663	SAMN03431378	SAMN02677678	SAMN20248551		SAMN12610330	SAMN08134253	
SAMN03702261		SAMN06142591	SAMN07340517	SAMN02677679	SAMN09953932		SAMN12610365	SAMN03277649	
SAMN03702242		SAMN02923489	SAMN07340518	SAMN02677680	SAMN09953931		SAMN02769705	SAMN03277691	
SAMN17188176		SAMN02923382	SAMN07340520	SAMN02677681	SAMN09953934		SAMN02769705	SAMN03277690	
SAMN18800702		SAMN02870507	SAMN07340527	SAMN02677682	SAMN09953955		SAMN22060992	SAMN03277690	
SAMN16814128		SAMN02922395	SAMN07340516	SAMN02677683	SAMN10083725		SAMN22060940	SAMN06240113	
SAMN16816223		SAMN02937502	SAMN02921967	SAMN02677686	SAMN15913291		SAMN02769789	SAMN03277693	
SAMN16818241		SAMN02923307	SAMN13013045	SAMN02850676	SAMN15913284		SAMN02769788	SAMN03277698	
SAMN16825942		SAMN02922492		SAMN04126428	SAMN15913283		SAMN02769790	SAMN03354303	
SAMN16825959		SAMN02923321		SAMN02677687	SAMN15970341		SAMN02769791	SAMN03354307	
SAMN16826279		SAMN02923320		SAMN02677688	SAMN15970340		SAMN03354304	SAMN03277703	
SAMN16826283		SAMN02923317		SAMN02677689	SAMN15970338		SAMN02769793	SAMN03277672	
SAMN16826324		SAMN02923319		SAMN03285248	SAMN15970337		SAMN02769792	SAMN06240110	
SAMN16826478		SAMN02923318		SAMN03285247	SAMN15970353		SAMN02769794	SAMN11268715	
SAMN16946591		SAMN02922493		SAMN03285250	SAMN15970352		SAMN02769778	SAMN03277704	
SAMN18800691		SAMN02937500		SAMN03285249	SAMN20248550		SAMN02769776	SAMN02769777	
SAMN18800681		SAMN02923306		SAMN02850684	SAMN10083729		SAMN03277683	SAMN05371163	
SAMN16816007		SAMN02923305		SAMN03354356	SAMN10026590		SAMN03277676		
SAMN16814148		SAMN02923304		SAMN02689388	SAMN15913280		SAMN08134158		
SAMN16825976		SAMN02923302		SAMN02689388	SAMN09953937		SAMN09756855		
SAMN16826090		SAMN02923303		SAMN02923022	SAMN09953940		SAMN06240101		
SAMN16814346		SAMN02923301		SAMN02923023	SAMN10026579		SAMN06240098		
SAMN16815308		SAMN02923300		SAMN02923025	SAMN10026602		SAMN03277713		
SAMN16822840		SAMN02923299		SAMN02923024	SAMN10026600		SAMN09756938		
SAMN16826314		SAMN02922491		SAMN02923026	SAMN10083726		SAMN09756990		
SAMN16826841		SAMN06446243		SAMN02923027	SAMN15913257		SAMN08134176		
SAMN15508338		SAMN02709176		SAMN02923021	SAMN15913298		SAMN14424504		
SAMN16813071		SAMN02921926		SAMN02420421	SAMN15913293		SAMN06240096		
SAMN16814577		SAMN02921925		SAMN02420422	SAMN15913256		SAMN03277774		
SAMN16817060		SAMN02709221		SAMN07204969	SAMN15913289		SAMN06240107		
SAMN16822767		SAMN02921698		SAMN04621511	SAMN15913287		SAMN09756905		
SAMN17188254		SAMN02921699		SAMN05967929	SAMN15913286		SAMN09756940		
SAMN17188262		SAMN03140285		SAMN14901720	SAMN15913285		SAMN02389791		
SAMN17188501		SAMN05191809		SAMN14901719	SAMN15913282		SAMN02389113		
SAMN16812873		SAMN06561800		SAMN03285252	SAMN15913278		SAMN02389111		
SAMN16826480		SAMN03140284		SAMN03285253	SAMN15913277		SAMN02381993		
SAMN17188259		SAMN03140286		SAMN03285254	SAMN15913275		SAMN09756899		
SAMN17015185		SAMN05191808		SAMN03285255	SAMN15913272		SAMN09756901		
SAMN16815988		SAMN13059119		SAMN03285256	SAMN15913270		SAMN02389108		
SAMN16817003		SAMN13059131		SAMN04437620	SAMN15913269		SAMN02389792		
SAMN16816250		SAMN13059120		SAMN04437622	SAMN15913266		SAMN09756948		
SAMN16826058		SAMN13059121		SAMN04437623	SAMN15970342		SAMN02389109		
SAMN17188241		SAMN13059593		SAMN04437621	SAMN15970358		SAMN09756896		
SAMN16816406		SAMN13059132		SAMN03285251	SAMN15970355		SAMN02389112		
SAMN16817036		SAMN13059130		SAMN03893567	SAMN15970351		SAMN09756946		
SAMN16826622		SAMN06561809		SAMN02769764	SAMN15970350		SAMN20504393		
SAMN16812857		SAMN06561811		SAMN02769763	SAMN15970349		SAMN20504392		
SAMN16814352		SAMN03702244		SAMN02769762	SAMN15970346		SAMN03277696		
SAMN16816407		SAMN03702240		SAMN15913295	SAMN20248557		SAMN08134186		
SAMN16821857		SAMN03702241		SAMN15913268	SAMN20248556		SAMN06240097		
SAMN17188564		SAMN08166092		SAMN15913253	SAMN20248552		SAMN07831758		
SAMN16813101		SAMN02352654		SAMN15970339	SAMN09953962		SAMN07831759		
SAMN16814437		SAMN08108723		SAMN20248555	SAMN09953944		SAMN09756862		
SAMN16826457		SAMN05521501		SAMN20248554	SAMN09953941		SAMN09756860		
SAMN17015077		SAMN05521500		SAMN10026598	SAMN09953935		SAMN09756859		
SAMN17188902		SAMN05521504		SAMN10083720	SAMN09953939		SAMN09756842		
SAMN15508303		SAMN05521503		SAMN10083737	SAMN10083719		SAMN09756861		
SAMN15508315		SAMN05521502		SAMN10083739	SAMN10083732		SAMN06240105		
SAMN16826244		SAMN05521499		SAMN15913292	SAMN15970336		SAMN03354316		
SAMN16826282		SAMN05384833		SAMN15913271	SAMN15970344		SAMN03354317		
SAMN16814153		SAMN03736725		SAMN15913296	SAMN09953945		SAMN03354315		
